# KRAS mutated Non‐Small Lung Carcinoma: A Real World Context from the Indian subcontinent

**DOI:** 10.1002/cam4.5193

**Published:** 2022-09-07

**Authors:** Ullas Batra, Shrinidhi Nathany, Mansi Sharma, Amrith BP, Joslia T. Jose, Harkirat Singh, Sakshi Mattoo, Anurag Mehta

**Affiliations:** ^1^ Medical Oncology Rajiv Gandhi Cancer Institute and Research Center New Delhi India; ^2^ Molecular Diagnostics Rajiv Gandhi Cancer Institute and Research Center New Delhi India; ^3^ Laboratory Services Rajiv Gandhi Cancer Institute and Research Center New Delhi India

**Keywords:** G12C, India, KRAS, NSCLC, real‐world

## Abstract

**Background:**

*KRAS*, although a common variant of occurrence (~20% of non‐small‐cell lung carcinoma [NSCLC]) has been untargetable, owing to the molecular structure which inherently prevents drug binding. *KRAS* mutations in NSCLC are associated with distinct clinical profiles including smokers and mucinous histology. KRAS G12C mutations account for ~40% KRAS altered NSCLC, but NSCLC being a geographically diverse disease, the features may be distinct in this part of the world. This is a single‐center experience of *KRAS*‐mutated NSCLC including clinical, imaging, pathologic features, and treatment patterns and outcomes.

**Methods:**

This is a single‐center retrospective study of KRAS‐mutated NSCLC. The clinicopathological features and outcomes were retrieved and collated from the medical record archives of the hospital.

**Results:**

Fifty (30.6%) patients with advanced‐stage NSCLC with alterations in the *KRAS* gene were enrolled in the 163 patients who were tested for *KRAS* alterations. The median age was 61 years. Molecular detection revealed three main types of *KRAS* mutations viz‐a‐vis: G12C in 17 (34%), G12V in 9 (18%), and G12D in 6 (12%) patients. Comparing G12C versus the non‐G12C mutated cases, co‐mutations were common in the non‐G12C subgroup (*p* < 0.05). Among the 36, who were treated at our center, all received chemotherapy as the first line with a median progression‐free survival (PFS)of 5.4 months. The PFS of G12C was higher than the non‐G12C subgroup (6.4 vs 3.8 months).

**Conclusion:**

This is the largest single‐center experience from the Indian subcontinent for KRAS‐mutated NSCLC with distinct clinical features. It highlights the unmet need for G12C inhibitors in our country, where prevalence is equivalent to the West.

## Graphical abstract

Real world study depicting prevalence of KRAS mutated NSCLC (30.6%) in India with G12C seen in 34% cases. Co‐mutations were higher in non G12C subgroup (p<0.05), and the PFS with chemotherapy was longer in G12C subgroup (6.4 months vs. 3.8 months).

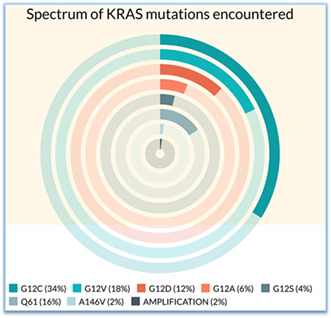

## 1 INTRODUCTION

Advanced metastatic non‐small‐cell lung carcinoma (NSCLC) has witnessed a dramatic paradigm over the last decade owing to the development and approvals of targeted therapy and immunotherapy. The most important breakthroughs despite the ongoing Covid‐19 pandemic include the incorporation of *MET (*Mesenchymal Epithelial Transition factor), *RET (*Rearranged during transfection), and *KRAS* (Kirsten Rous sarcoma virus) in biomarker testing for NSCLC as well as the recent recommendation of performing broad panel‐based testing in patients of squamous histology as well.^1^, ^2^

RAS gene was identified in the 1980s (1986) and is one of the most common genes implicated in common malignancies.^3^ There are three isoforms: KRAS, NRAS, and HRAS, among which KRAS is the one that is implicated in lung cancer oncogenesis. So long considered undruggable,^4^, ^5^, ^6^, ^7^ evolution of molecular biology has led to one of the most important developments. The approval of *KRAS* G12C (G‐glycine, C‐cysteine) inhibitor, sotorasib^8^, ^9^ for use in the second‐line setting, has revolutionized the treatment of this subgroup of NSCLC patients. For almost two to three decades, *KRAS*, although a common variant of occurrence (~20% of NSCLC) has been untargetable, owing to the molecular structure which inherently prevents drug binding. The G12C residue allows the binding of covalent inhibitors and in turn causes allosteric inhibition of the 12‐cysteine.^8^ This discovery led to a series of trials including CodeBreak 100,^10^ 200,^11^ etc., which have shown initial promising results, culminating in rapid Food and Drug Administration (FDA) approval of sotorasib.

*KRAS* mutations in NSCLC are associated with distinct clinical profiles including smokers and mucinous histology and have been studied in large cohorts worldwide.^12^ The specific clinical, pathologic features, and treatment outcomes have been described in a few real‐world studies and in controlled trials. However, real‐world data from the Indian subcontinent and analogous to other biomarker‐driven processes in NSCLC such as *EGFR* (Epidermal growth factor receptor), *ALK* (Anaplastic lymphoma kinase), and *KRAS* also show some distinct differences from the Western population, hence warrants comprehensive reporting of these findings.

KRAS G12C mutations account for ~40% of KRAS altered NSCLC,^13^ but NSCLC being a geographically diverse disease, the features may be distinct in this part of the world.

This is a single‐center experience of *KRAS*‐mutated NSCLC including clinical, imaging, pathologic features, and treatment patterns and outcomes. Although sotorasib access is restricted currently to the West, it is important to report the epidemiology of this subgroup in order to understand the urgent need for the availability of the drug in this part of the world.

## 2 MATERIALS AND METHODS

### 2.1 Patients

Among the 3899 NSCLC patients attending the outpatient facility of Rajiv Gandhi Cancer Institute and Research Center between 2016 and 2020, 163 patients were tested for *KRAS* alterations (the remaining patients could not be tested due to economic restraints, as KRAS alterations were tested using next‐generation sequencing technology), of which 50 patients have confirmed to harbor a *KRAS* variant by molecular testing. The patients with biopsy‐proven NSCLC, irrespective of the type of histology, and stage were included. Patients with metastases to the lung, small‐cell carcinoma, and unavailability of biopsy reports were excluded from the study. For the analysis of KRAS alterations, patients who were KRAS‐mutated (harbored a mutation in the KRAS gene, proved on NGS) were included. The clinical features, imaging and pathological diagnoses, and staging as well as alterations detected on molecular testing were recorded. The treatment modalities and responses were recorded. The histologic subtyping was determined according to the 2021 World Health Organization classification. The response to treatment was evaluated in accordance with the Response Evaluation Criteria in Solid Tumors (RECIST version1.1).^14^ This study was approved by the Institutional review board (vide no. RGCIRC/IRB‐BHR/114/2020). The STROBE checklist has been used for reporting the results (Supplementary file).

### 2.2 Molecular detection

#### 2.2.1 Next‐generation sequencing (NGS)

Both DNA‐ and RNA‐based NGS testing were done using the Oncomine Focus Assay panel which encompasses 52 genes including single‐nucleotide variations, indels (insertions and deletions), fusions, and copy number alterations across various solid organ malignancies. The details of the performance and assay methodology can be found in our study published earlier.^15^ In brief, Oncomine Focus Assay (Thermofisher Scientific, CA, USA) (comprising 52 genes, interrogating for single‐nucleotide variations, indels, fusions, and copy number alterations) was used. DNA and RNA were extracted from formalin‐fixed paraffin‐embedded (FFPE) tissue blocks, and quality and quantity were checked using TapeStation (Agilent). Libraries were prepared and run on the Ion Torrent S5 platform. The reports were generated using Oncomine Knowledge Reporter Software after the assessment of optimum quality metrics. The variants were called and visualized on Integrative Genomics Viewer to ascertain the validity of the call.

### 2.3 Survival analysis

The overall survival (OS) was defined as the period from the date of diagnosis until the date of death. For patients who were alive at the time of the final follow‐up date, survival was censored at the date of the last visit of follow‐up. The progression‐free survival (PFS) was defined as the period from the date of initiation of first‐line therapy until the date of progression/change of drug whichever occurred earlier.

### 2.4 Statistical analysis

All statistical analyses were performed with SPSS version 23 (IBM Corporation, Armonk, NY, USA) and MedCalc (Ostend, Belgium). Categorical variables were depicted and presented in frequencies with their respective percentages. The differences between *KRAS* G12C mutant and non‐G12C mutant were compared using Fisher's exact test or Chi‐squared test with a two‐sided *p* value of <0.05 being considered significant. Survival analysis was done using Kaplan–Meier method, and the two‐sided log‐rank test was used for univariate survival analysis.

## 3 RESULTS

### 3.1 Patient characteristics and treatment details

A total of 50 (30.6%) patients with advanced‐stage NSCLC with alterations in the *KRAS* gene were enrolled in the 163 patients who were tested for *KRAS* alterations. The median age was 61 years (range:31–86 years). There were 32 males (64%) and 18 females (36%), with a male to female ratio being 1.8:1. Thirty‐nine patients (78%) depicted adenocarcinoma histology, 5 (10%) depicted squamous histology with moderate differentiation, 2 (4%) patients had sarcomatoid, and 4 (8%) showed not otherwise specified type morphology. Among those with squamous histology all 5 patients were smokers, and 12 with adenocarcinoma histology had a history of smoking. With respect to metastases to the brain, 12 patients had metastases to the brain at diagnosis, and 2 developed later during the course of their disease. PDL1 testing (SP263, Ventana) revealed >50% in 8 patients. The detailed baseline characteristics of G12C positive vs negative are depicted in Table 1.

**TABLE 1:** Comparison between G12C and non‐G12C‐mutated groups with respect to clinical features and outcomes

Clinical features	G12C‐%	Non‐G12C‐%	*p* value
Age, median	63	63	
Gender			
Male	10 (58.8)	17 (51.5)	0.1
Female	7 (41.2)	16 (48.5)	
Smoker	16 (94.1)	4 (12.1)	0.09
Never smoker	1 (5.9)	29 (87.9)	
PDL1			
<1%	4 (23.5)	18 (54.5)	0.09
1–49%	11 (64.7)	9 (27.3)	
≥50%	2 (11.8)	6 (18.2)	
Brain mets at diag			
Present	5 (29.4)	6 (18.2)	0.07
Absent	12 (70.6)	27 (81.8)	
PFS on chemotherapy	6.4 months (95% CI: 2.8–11.8)	3.8 months (95%CI: 2.9–7.7)	0.08

Abbreviations: *diag*, diagnosis; *mets*, metastases; PDL1, programmed death ligand 1; PFS, progression‐free survival,

### 3.2 Spectrum of KRAS mutations

**FIGURE 1:**
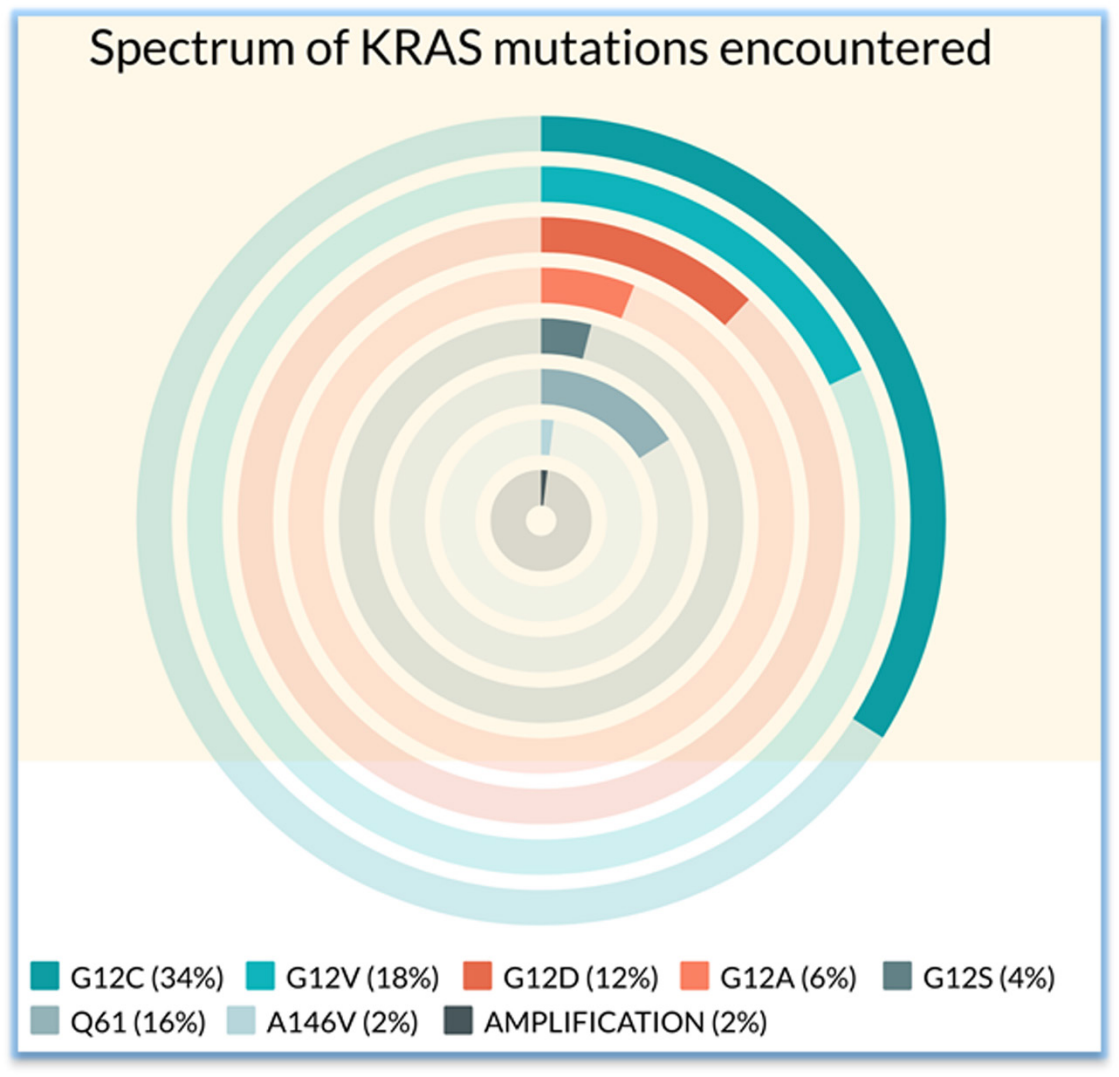
Plot showing the frequencies of occurrence of subtypes of KRAS mutations encountered in the study population. G12C in 17 (34%), G12V in 9 (18%), and G12D in 6 (12%) patients. The other mutations detected were G12A (3, 6%), G12S (2, 4%), Q61H/K/L (8, 16%), and A146V (1, 2%), respectively. *KRAS* copy number gain with 12.8 copies was detected in 1 (2%) case.

Molecular detection revealed three main types of *KRAS* mutations viz‐a‐vis: G12C in 17 (34%), G12V in 9 (18%), and G12D in 6 (12%) patients. The other mutations detected were G12A (3, 6%), G12S (2, 4%), Q61H/K/L (8, 16%), and A146V (1, 2%). *KRAS* copy number gain with 12.8 copies was detected in 1(2%) case. The types of KRAS alteration detected with their frequencies are depicted in Figure 1. None of the patients had *KRAS* complex mutations. With respect to the relationship between PDL1 expression and type of *KRAS* alteration, *KRAS* G12C subgroup showed lower PDL1 expression compared with the non‐G12C group, although the values did not reach statistical significance.

### 3.3 Concurrent mutations in other tumor suppressors and oncogenes

**FIGURE 2:**
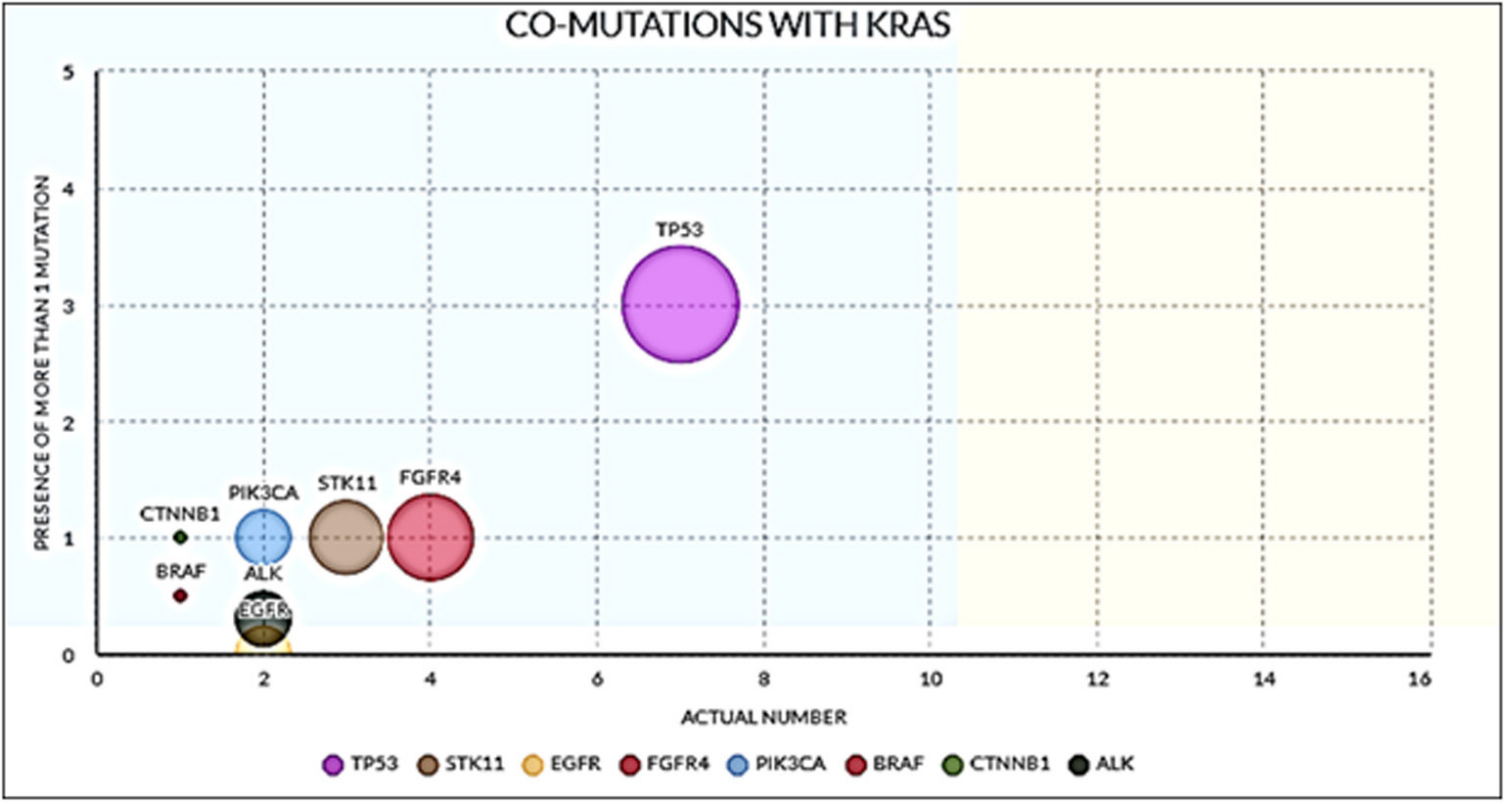
Plot showing the relative frequencies of co‐mutations encountered in the KRAS‐mutated cohort. The predominant co‐mutations that were detected included *TP53* in 7 (14%); *STK11* in 3 (6%); *FGFR4* in 4 (8%); *PIK3CA, EGFR*, and *ALK* in 2 cases each (4% each); and *BRAF* and *CTNNB1* in case each (2% each).

The predominant co‐mutations which were detected included *TP53* in 7 (14%); *STK11* in 3 (6%); *FGFR4* in 4 (8%); *PIK3CA, EGFR*, and *ALK* in 2 cases (4% each); and *BRAF* and *CTNNB1* in case each (2% each). Comparing G12C versus the non‐G12C‐mutated cases, co‐mutations were common in the non‐G12C subgroup (*p* < 0.05). The percentage and frequencies of the co‐mutations detected are depicted in Table 2 and Figure 2.

**TABLE 2:** The frequencies of occurrence of co‐mutated genes in the study population

Gene	*N* (%)
*TP53* *	7 (14)
*STK11* *	3 (6)
*FGFR4*	4 (8)
*PIK3CA*	2 (4)
*BRAF*	1 (2)
*CTNNB1*	1 (2)
*EGFR*	2 (4)
*ALK*	2 (4)

^a^ Tested in 22 cases only.

### 3.4 Treatment and follow‐up

Among the 50 patients, 36 patients received treatment, whereas 14 were lost to further follow‐up. The cases with other driver alterations like *EGFR* and *ALK* were also excluded from this analysis, as *KRAS* alteration would have plausibly occurred as a resistance mechanism to the targeted treatment administered to these patients. Among the 36 who were treated at our center, all received chemotherapy as the first line with a median PFS of 5.4 months. The PFS of G12C was higher than the non‐G12C subgroup (6.4 vs 3.8 months). The median follow‐up time was 14.3 months. The median OS of the entire cohort was 11.1 months (95% CI: 6–18). There was no statistically significant difference in OS between KRAS G12c vs the non‐G12C group treated with chemotherapy (15.2 months vs 16.1 months, *p* = 0.2). Owing to the small sample size of patients who underwent extended molecular profiling for concurrent mutations, this finding has to be interpreted with caution. Three patients were offered immune checkpoint inhibitors (ICI) in the form of nivolumab therapy after progression on chemotherapy. The PFS for nivolumab was 3.6 months, 4.6 months, and 5 months, respectively, in the three cases. The number in this section of ICI‐treated cases is limited for any further analysis.

## 4 DISCUSSION

This is a single‐center real‐world experience of KRAS‐mutated NSCLC from the Indian subcontinent.

Among those who underwent testing, 50 (30.6%) cases were found to be *KRAS* mutated, which is comparable to reported contemporary literature (25–35%), although Nacchios et al.^16^ reported a lower frequency of 18.6%. Cui et al.^17^ reported a frequency of 42%. The most common variant was G12C seen in 34% of cases. This is comparable to the study by Nacchios et al.,^16^ which also depicted G12C to be the most common variant seen at 36.1%, whereas Cui et al.,^17^ reported a high percentage of 45%. Aredo et al.,^18^ reported a prevalence of 35% of G12C among 186 patients enrolled in their study. Our finding was concordant with Lei et al who reported G12C in 33% of cases as well as Aredo et al. (35%).^18^ These differences in prevalence may be attributed to differences in sample size, apart from geographic and ethnic differences.

With respect to the clinical profile, it has been reported that KRAS alterations occur more commonly in smokers or ex‐smokers.^19^ Additionally, it is well documented that among the different types of *KRAS* mutation, transition mutations resulting in G12D are common in nonsmokers compared to transversion mutations like G12C, which has been noted in the current study.^13^ Dogan et al.^20^ reported in their study that G12C mutation was more frequently seen in women compared with other mutations, however, this difference was not demonstrated in our study. Cui et al.^17^ reported a frequency of 28% for patients diagnosed with brain metastasis at diagnosis, and 40% who developed later during the course of the disease in the G12C group. In the non‐G12C group, the frequencies reported were 19% and 41%, respectively, demonstrating that there is no actual difference. In our current study, the frequency of occurrence was 29.4% at diagnosis for the G12C group, in contrast to 18.2% in the non‐G12C group. The presence of co‐mutations like *TP53, STK11, KEAP1*, and *CDKN2A* has been widely studied, however, in our study, the numbers in each group (TP53 and STK11) are too small for separate analyses. CDKN2A and KEAP1 were not sequenced as a part of the genomic profiling panel at our center.^12^, ^21^, ^22^, ^23^

Prognostically, a meta‐analysis in 2013^24^ which included 1543 patients showed no difference in OS in *KRAS*‐mutant and *KRAS*‐wild‐type groups in early stage disease. In the advanced stage, however, which included 6939 patients,^24^, ^25^ the *KRAS*‐mutant group showed a poor OS compared with wild‐type counterparts. Svaton et al.,^26^ in a real‐world experience of 39 patients, demonstrated a shorter OS for the G12C group compared with other *KRAS* mutations, whereas in another study on 677 patients, Yu and Sima et al.^27^ demonstrated no difference in OS between the two subgroups. However, this study did not include KRAS wild‐type patients. In our study also, there was no statistical difference in OS between the two subgroups. However, overall PFS and OS are not represented here in graphical form, owing to the limited sample size in the study. The median PFS of those treated with chemotherapy was 5.4 months with G12C demonstrating a higher PFS compared with non‐G12C patients. This is in concordance with Lei et al.,^28^, ^29^ who reported the first‐line PFS of 5 months, and a higher PFS for G12C when compared with the non‐G12C group (4.7 months vs 2.5 months). Minor differences may be attributed to sample size and statistical bias introduced due to the presence of confounding factors like age, brain metastases, etc.

## 5 CONCLUSION

This is by far the largest experience from India of *KRAS*‐mutated NSCLC, comparing G12C with other KRAS mutants. Despite limitations of retrospective nature, small sample size, heterogeneity in the cohort limited survival analysis due to missing data points, and limited molecular profiling this study is relevant in the light of the approval of targeted therapy, sotorasib, which is currently unavailable in this country.

## DUAL PUBLICATION STATEMENT

The manuscript has not been published/submitted/under consideration in any other journal.

## AUTHOR CONTRIBUTIONS

UB: conceptualization, Clinical Review, Manuscript writing, and finalization of draft.

SN: Data curation, analysis and manuscript writing, molecular testing.

JTJ and HKS: Data curation and patient follow‐up.

MS and ABP: Clinical review of patients and manuscript review.

SM: Molecular diagnostics.

AM: Pathologic review and Molecular reporting.

## FUNDING INFORMATION

None.

## Supporting information


Appendix S1

Appendix S2

## Data Availability

The data that support the findings of this study are available from the corresponding author upon reasonable request.
