# Supplementary figures and images for: KRAS mutated Non‐Small Lung Carcinoma: A Real World Context from the Indian subcontinent
Source: Cancer Med. 2022 Sep 7;12(3):2869–74. doi: 10.1002/cam4.5193 (PMC9939094; doi:10.1002/cam4.5193)

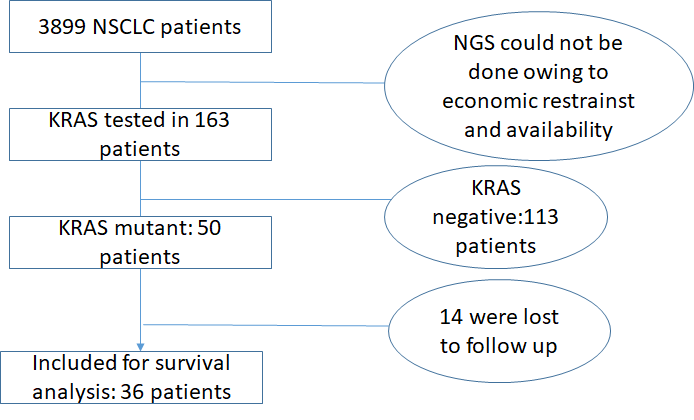


Supplementary: Flow diagram showing recruitment and attrition of patients

Supplement: Supplementary file 2 — Appendix S2 [file CAM4-12-2869-s002.docx]
